# Evaluating the cost-effectiveness of a pre-exposure prophylaxis program for HIV prevention for men who have sex with men in Japan

**DOI:** 10.1038/s41598-022-07116-4

**Published:** 2022-02-23

**Authors:** Nao Yamamoto, Yoshiki Koizumi, Shinya Tsuzuki, Keisuke Ejima, Misao Takano, Shingo Iwami, Daisuke Mizushima, Shinichi Oka

**Affiliations:** 1 School of Human Evolution and Social Change, Arizona State University, Tempe, USA; 2 National Center for Global Health and Medicine AIDS Clinical Center, Tokyo, Japan; 3 National Center for Global Health and Medicine AMR Clinical Reference Center, Tokyo, Japan; 4 Faculty of Medicine and Health Sciences, University of Antwerp, Antwerp, Belgium; 5 Department of Epidemiology and Biostatistics, Indiana University School of Public Health-Bloomington, Bloomington, USA; 6 Division of Biological Science, Graduate School of Science, Nagoya University, Nagoya, Japan; 7 MIRAI, JST, Saitama, Japan; 8 Nstitute for the Advanced Study of Human Biology (ASHBi), Kyoto University, Kyoto, Japan; 9 NEXT-Ganken Program, Japanese Foundation for Cancer Research (JFCR), Tokyo, Japan; 10 Science Groove Inc., Fukuoka, Japan

**Keywords:** Disease prevention, Health care economics, Public health

## Abstract

Men who have sex with men (MSM) have been disproportionally affected by the HIV epidemic in many countries, including Japan. Although pre-exposure prophylaxis (PrEP) is a strong prevention tool, it is not yet approved in Japan. A Markov model was developed to describe HIV infection and disease progression in an MSM cohort (N = 1000) in Japan receiving a PrEP program. The model was used to evaluate the cost-effectiveness of a PrEP program. HIV/AIDS treatment, screening, hospitalization due to AIDS, and PrEP were considered as costs and quality-adjusted life-years (QALYs) gained as utilities. Cost-effectiveness was assessed by comparing the incremental cost-effectiveness ratio (ICER) over a 30-year period against the willingness to pay (WTP) threshold. One-way sensitivity and probabilistic sensitivity analyses were performed. With 50% PrEP coverage, the PrEP program became dominant against the program without PrEP, using a threshold of 5.0 million JPY/QALY (45,455 USD). The probabilistic sensitivity analysis revealed that the PrEP program was dominant or at least cost-effective in most cases of 10,000 simulations. Therefore, preparing cheaper PrEP pills, which results in PrEP being dominant or ICER being lower than the WTP threshold, is important to make the program cost-effective. Introduction of PrEP to an MSM cohort in Japan would be cost-effective over a 30-year time horizon.

## Introduction

HIV/AIDS is not yet a curable disease. However, the expected lifespan of those living with HIV infection and receiving antiretroviral therapy (ART) in high-income countries, especially with early initiation of treatment, is now comparable with that among people without HIV^1,2^. In addition, the transmission risk among people who have successfully achieved and maintained viral suppression through ART is negligibly low^3–5^. Therefore, ART is considered both a treatment tool for those living with HIV and a prevention tool (i.e., “treatment as prevention”). Based on such scientific evidence, the Undetectable = Untransmittable (U = U) campaign has spread worldwide^6,7^ to encourage people at risk for HIV infection to undergo routine testing for early diagnosis and treatment initiation if necessary.

Furthermore, ART is now also used for prophylactic purposes. People who are HIV-negative can avoid infection by taking ART before or after possible exposure. Taking ART before exposure is called pre-exposure prophylaxis (PrEP). The first PrEP treatment using antiretroviral drugs (tenofovir/emtricitabine) was approved by the US Food and Drug Administration in 2012. The World Health Organization (WHO) published guidelines on the use of PrEP in 2015 and recommended it as a prevention choice for people at substantial risk for HIV infection, such as men who have sex with men (MSM)^8,9^. Since then, many countries have approved antiretroviral drugs for PrEP use^10^.

When considering PrEP for approval, a crucial question concerning its use for HIV prevention is whether it is cost-effective from a healthcare payer perspective; that is, formal healthcare (medical) costs borne by third-party payers or paid for out-of-pocket by patients^11,12^. HIV incidence is central to determining cost-effectiveness. For example, the WHO and the International Antiviral Society-USA recommend PrEP for populations with an HIV incidence of 3.0% or 2.0% per year or higher^8,9^. UNAIDS did not specify a threshold for incidence, but stated PrEP is only cost-effective for those at high risk^13^. Several cost-effectiveness analyses of PrEP introduction have been conducted in various countries, including the UK, the Netherlands, and the US^14–17^. The findings of these studies were consistent with the UNAIDS statement.

Although the introduction of PrEP has been discussed in Japan, it is not yet approved. A potential barrier to PrEP approval in Japan may be that the PrEP program is not considered cost-effective because the incidence and prevalence of HIV are lower than in other high-income countries (i.e., the cost of the PrEP program exceeds the benefit gained by the program). Even among MSM in Japan, which account for nearly 72.3% of the incidence of HIV infection in the country^18^, the HIV incidence is 3.8% per year, which is close to the 3.0% threshold^18–21^. Furthermore, given that costs of PrEP and HIV/AIDS treatment in Japan are higher than in other counties where cost-effectiveness studies have been performed^22^, cost-effectiveness analysis reflecting the situation in Japan is necessary to argue for PrEP approval.

The present study aimed to assess the cost-effectiveness of introducing a PrEP program to a cohort of MSM in Tokyo, Japan, where the risk for HIV infection is relatively low. We first developed a Markov model describing HIV infection and disease progression in a PrEP program over time in a MSM cohort. We assessed the cost-effectiveness of the PrEP program by comparing the cost of treatment and the PrEP program versus the utilities gained by the program.

## Results

**Figure 1:**
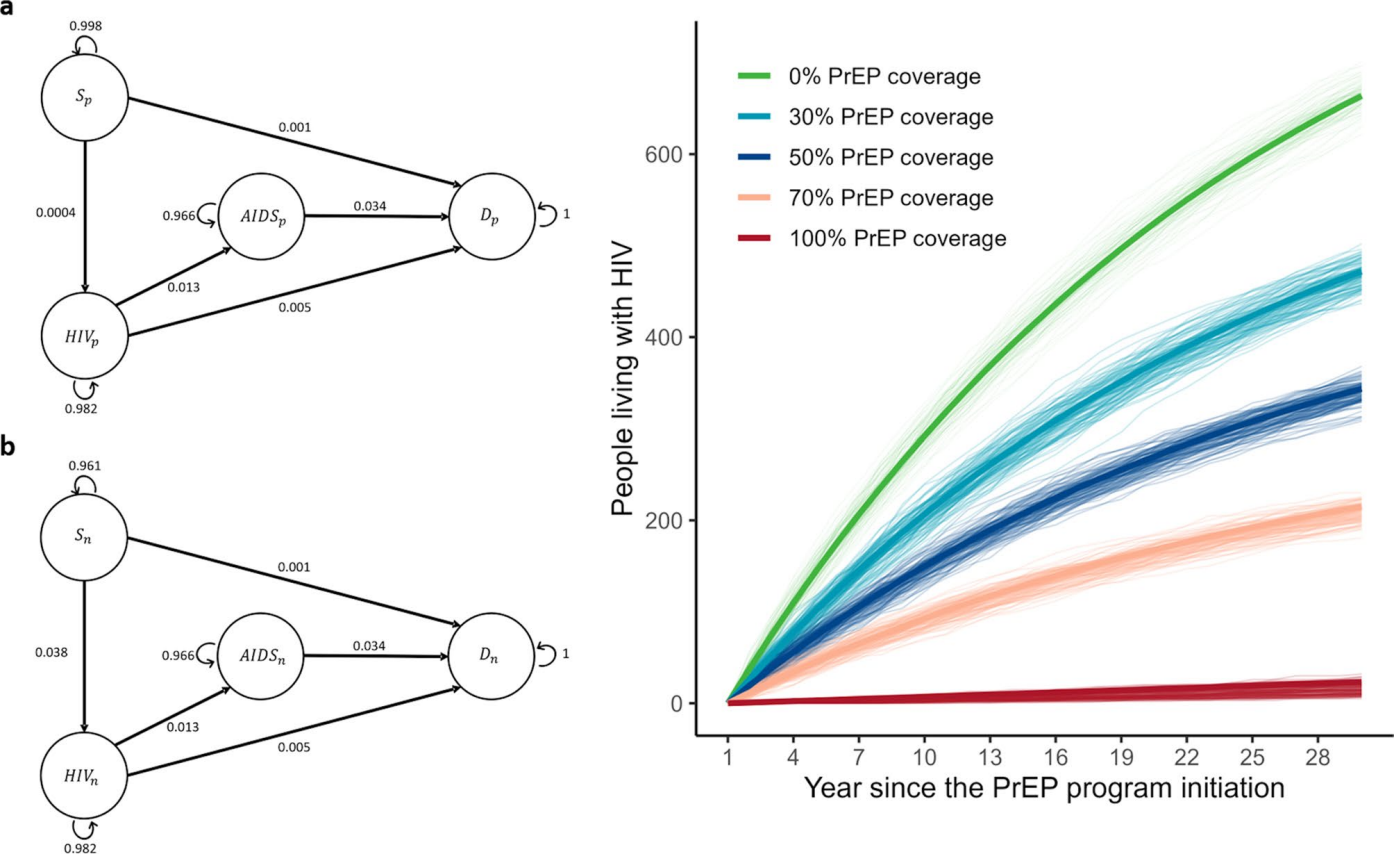
Schematic representation of the Markov model and the impact of pre-exposure prophylaxis (PrEP) on HIV incidence over 30 years. (**a**) The Markov model for individuals without PrEP (a-1) and with PrEP (a-2). Four different health status groups of infection and disease progression were considered: susceptible and uninfected (denoted by *S*), HIV-infected without AIDS (*HIV*), HIV-infected with AIDS (*AIDS*), and dead (*D*). The four health conditions were further annotated by PrEP status (e.g., *S* with and without PrEP denoted by *S*_*p*_ and *S*_*n*_, respectively). Arrows represent transitions between different states, and the numbers are transition probabilities. (**b**) Short-term impact of a PrEP program on the MSM cohort. The thin lines represent the results of 100 simulations for illustration purposes, and the solid lines represent the mean of 1000 simulations. The number of people living with HIV (i.e., individuals categorized in one of the four health status groups: $HI{V}_{p},HI{V}_{n},AID{S}_{p},AID{S}_{n}$) in a given year after initiation of the PrEP program.

The health status of infection and disease progression that have been modelled and presented here are described in further detail in the Methods (see Fig. 1a for a detailed schematic of the Markov model). Without the introduction of the PrEP program, the number of people living with HIV was 664 (interquartile range [IQR]: 633–693) after 30 years among 1,000 individuals (Fig. 1b). In the PrEP program (PrEP coverage of 30%, 50%, 70%, and 100% in the four groups), the number of people living with HIV was reduced by 192, 320, 449, 640 after 30 years, respectively. Because PrEP substantially reduced susceptibility to infection, HIV infection was rarely observed among people taking PrEP. Therefore, most infections observed were in people not taking PrEP.

**Figure 2:**
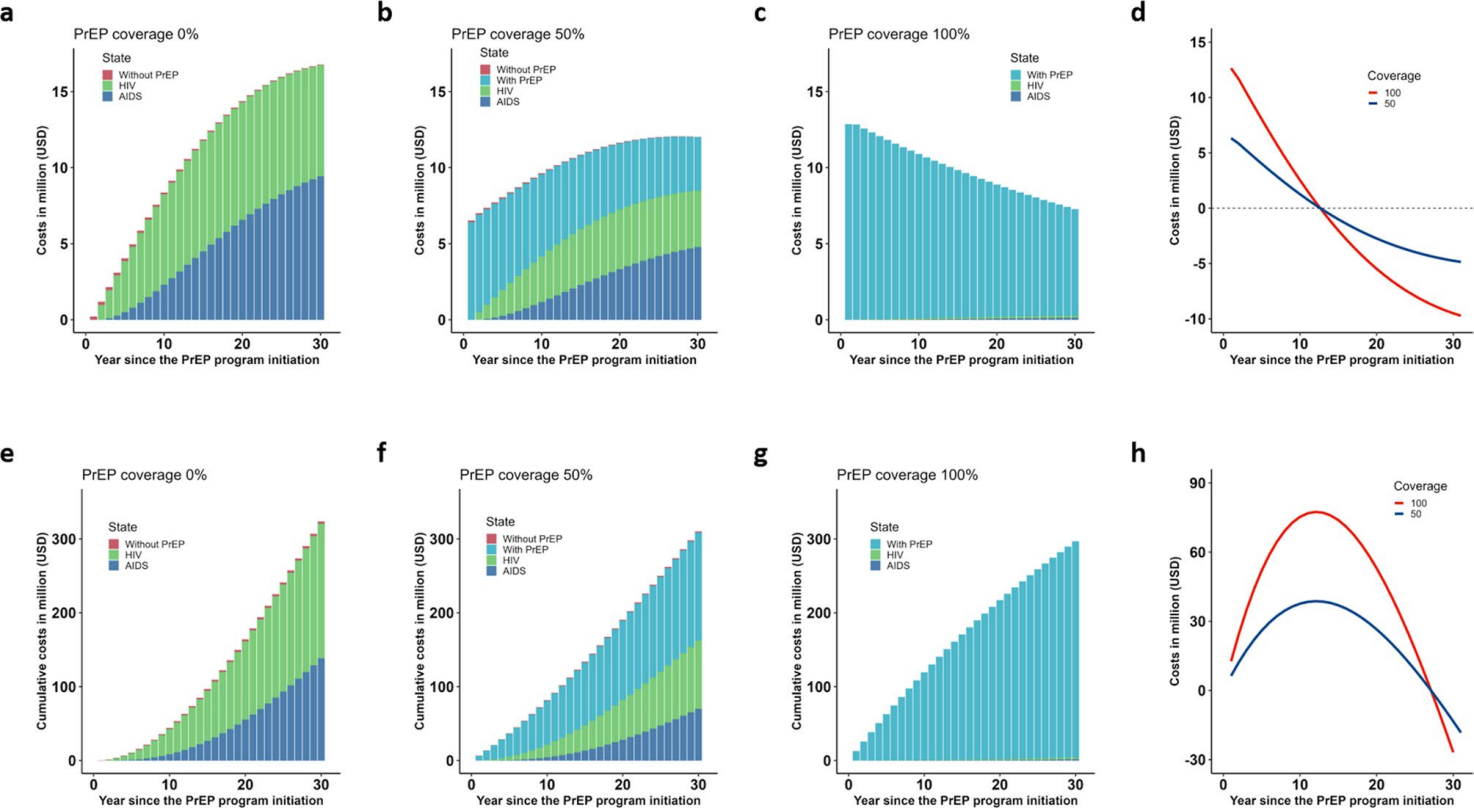
Yearly cost distribution over 30 years. Yearly costs related to treatment, screening, and pre-exposure prophylaxis (PrEP) were computed assuming an annual discount rate of 2%. (**a**) Yearly cost if PrEP is not introduced. (**b**) Yearly cost with the introduction of PrEP to 50% of the cohort. (**c**) Yearly cost with the introduction of PrEP to the whole cohort. (**d**) Difference in yearly cost between when PrEP is introduced with different coverages (50% or 100%) versus when PrEP is not introduced. (**e**) Cumulative cost if PrEP is not introduced. (**f**) Cumulative cost with the introduction of PrEP to 50% of the cohort. (**g**) Cumulative cost with the introduction of PrEP to the whole cohort. (**h**) Difference in cumulative cost between when PrEP is introduced with different coverages (50% or 100%) versus when PrEP is not introduced.

Figure 2a–d shows the yearly cost distributions over 30 years with and without PrEP after discounting. The cost was broken down into costs by status: susceptible status in the without-PrEP group (cost for screening); susceptible status in the with-PrEP group (cost for PrEP pills and screening); HIV status (cost for ART, screening, and health checkups); and AIDS status (cost for ART, screening, health checkups, and hospitalization). Without the PrEP program (Fig. 2a), the yearly cost increased over time and reached about 16.8 million USD (1,527.3 million JPY; 1 USD = 110 JPY) at year 30. 43.6% of this cost was accounted for by HIV status in the without-PrEP group, and 56.2% by AIDS status in the without-PrEP group. If PrEP was introduced to one-half of the cohort (i.e., 50% PrEP coverage; Fig. 2b), the cost for susceptible status in the with-PrEP group accounted for almost the total cost in the first few years. However, in year 30, it accounted for about 29.2% of the total cost of 12.0 million USD, because the cost for those with HIV (with or without AIDS) increased over time as more people were infected with HIV (mostly people in the without-PrEP group). If PrEP was introduced to the whole cohort (i.e., 100% PrEP coverage; Fig. 2c), the proportion of the cost for PrEP was almost 100% because PrEP mostly prevented new infection. The total cost decreased over time and reached about half of the cost of the first year (from 12.9 million USD to 7.3 million USD), but this was mainly because of discounting. In the first year, the total cost for the with-PrEP scenario was higher and the differences in cost between the scenario without PrEP and the scenarios with PrEP (50% or 100% coverage) were 6.5 million USD and 12.9 million USD, respectively. However, the difference between the total cost for the without-PrEP scenario and that for the with-PrEP scenarios decreased over time, and the sign flipped from plus to minus at year 13 when the total cost for the with-PrEP scenario was less than that for the without-PrEP scenario (Fig. 2d). The cumulative cost distributions over 30 years with and without PrEP after discounting are shown in Fig. 2e–h. Without the PrEP program (Fig. 2e), the cumulative cost reached about 323.6 million USD at year 30; 56.4% of this cost was accounted for by those with HIV without AIDS, and 42.7% by those with HIV with AIDS. If PrEP was introduced to half of the cohort (Fig. 2f), the cost for susceptible status in the with-PrEP group accounted for almost the total cost in the first few years. However, in year 30, this group accounted for 48.39% of the total cost of 310.2 million USD. If PrEP was introduced to the whole cohort, the proportion of the cost for PrEP was almost 100% of the total cost of 296.7 million USD in year 30. Differences in the cumulative cost between introduction of PrEP versus no introduction reached the maximum at year 13 and turned the sign flipped from plus to minus at year 28 (Fig. 2h). Moreover, the total cumulative costs of the scenario with PrEP became lower than the total costs of the scenario without PrEP at year 28.

**Figure 3:**
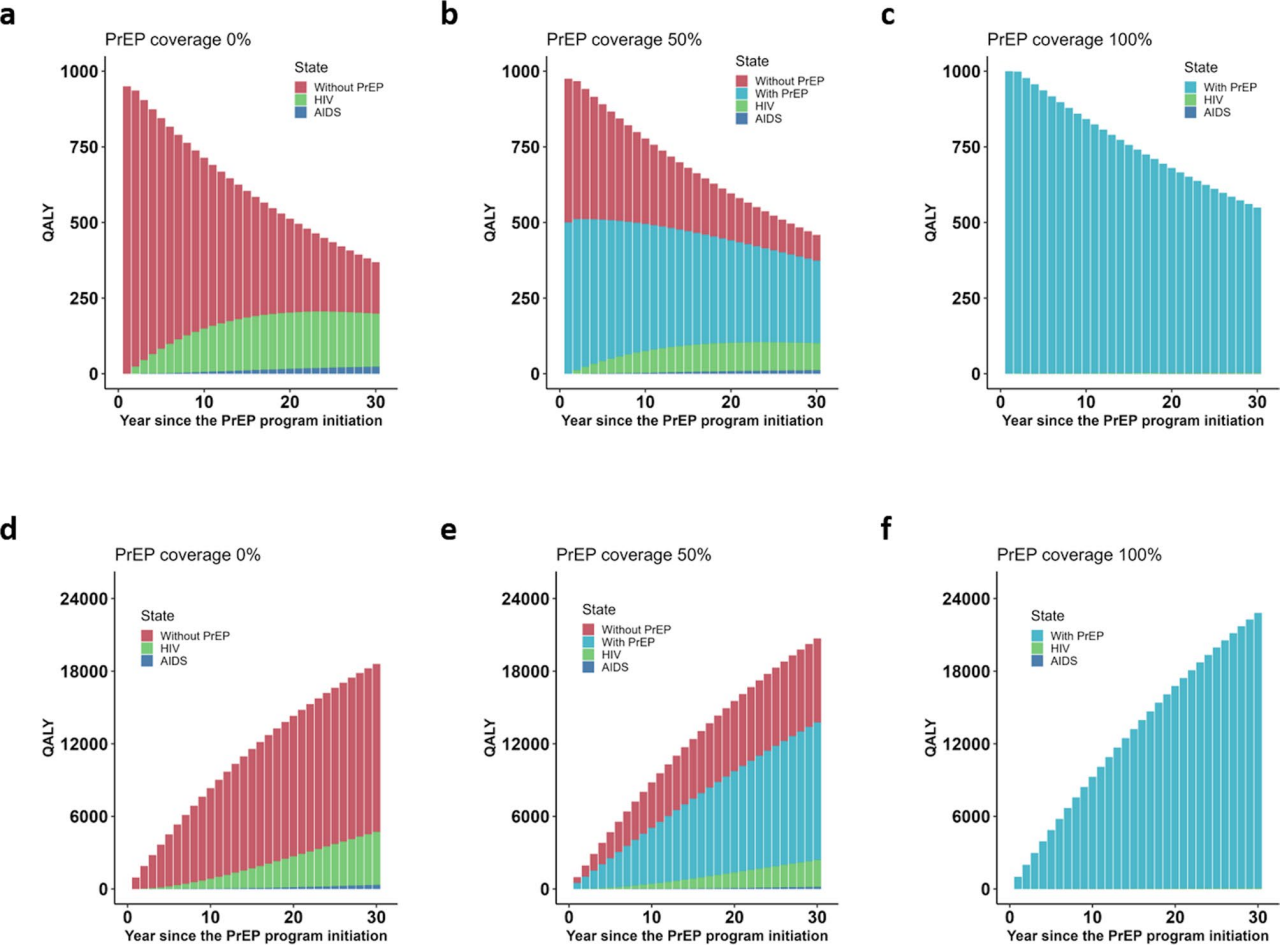
Yearly QALY distribution over 30 years. Yearly QALYs related to treatment and pre-exposure prophylaxis (PrEP) calculated, assuming an annual discount rate of 2%. (**a**) Yearly QALYs if PrEP is not introduced. (**b**) Yearly QALYs with the introduction of PrEP to 50% of the cohort. (**c**) Yearly QALYs with the introduction of PrEP to the whole cohort. (**d**) Cumulative QALYs if PrEP is not introduced. (**e**) Cumulative QALYs with the introduction of PrEP to 50% of the cohort. (**f**) Cumulative QALYs with the introduction of PrEP to the whole cohort.

Yearly quality-adjusted life-years (QALYs) were computed for each program (Fig. 3a–c**)**. The QALYs declined over time for all programs because QALYs assigned to those with susceptible status were larger than for other health status groups and because of discounting. Without the PrEP program (Fig. 3a), the yearly QALYs for susceptible status in the without-PrEP group decreased over time. QALYs for HIV status and AIDS status in the without-PrEP group slightly increased over time as the number of infected persons increased. As a result, QALYs for the whole population reached 38.8% of the initial QALYs at year 30. If PrEP was introduced to one-half of the cohort (Fig. 3b), the QALYs for susceptible status decreased over time and reached 47.1% of the initial QALYs. If PrEP was introduced to the whole cohort (Fig. 3c), QALYs only decreased because of discounting. The cumulative QALY distributions over 30 years with and without PrEP after discounting are shown in Fig. 3d–f. Without the PrEP program (Fig. 3d), the cumulative QALY reached about 18,597 at year 30. If PrEP was introduced to half of the cohort (Fig. 3e), the QALYs for 30 years reached 20,707. If PrEP was introduced to the whole cohort (Fig. 3f), the proportion of the QALYs for susceptible status in the with-PrEP group was almost 100%.

**Figure 4:**
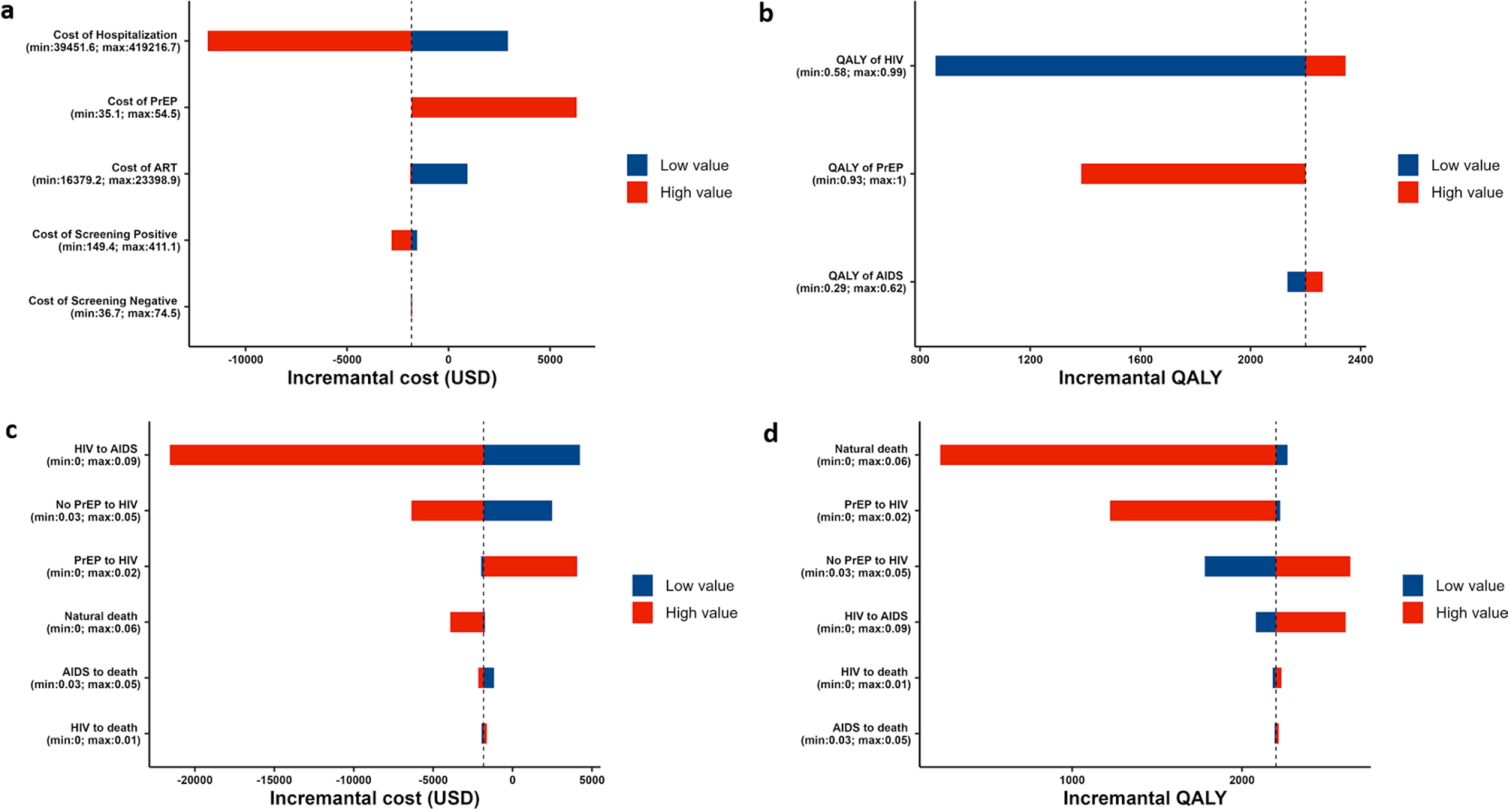
Cost-effectiveness evaluation of the pre-exposure prophylaxis (PrEP) program. The incremental cost and the incremental QALYs gained in the 30 years since the introduction of the PrEP program with varied parameter values was calculated. Incremental cost varying each cost, QALYs, or transition parameter. The black dashed vertical lines represent the base case scenario. Therefore, the values on the left of the base case scenario correspond to more favorable scenarios compared with baseline. Blue bars represent parameter values lower than baseline, and red bars represent parameter values higher than baseline. (**a**) Cost parameters were varied: the cost of PrEP pills, cost of hospitalization due to AIDS, cost of ART for those infected by HIV, cost of screening for the HIV-positive population, and cost of screening for the HIV-negative population. (**b**) QALY parameters varied: QALYs for people with PrEP, people living with HIV, and AIDS. (**c**) The transition probabilities of the Markov model also varied. For the transition probability from PrEP to HIV, the PrEP efficacy was varied.

With the baseline scenario of 50% PrEP coverage 28 years or longer after program initiation demonstrated negative value of incremental cost and positive value of QALYs gain, which shows that scenarios with longer time horizon are dominant against the without-PrEP program. Contrary, scenarios with time period for 13–27 years showed the incremental cost-effectiveness ratio (ICER) value smaller than our WTP. Figure 4a shows the one-way sensitivity analysis of the cost parameters on the cumulative cost for 30 years. The cost of hospitalization had the most influence on the incremental cost, followed by the cost of PrEP pills, whereas the cost of screening for susceptible and uninfected people and the cost of screening and health checkups for those with HIV barely influenced the incremental cost. Figure 4b shows the one-way sensitivity analysis for the QALY parameters. The QALYs for HIV status had the most influence on the incremental QALYs, whereas the QALYs for AIDS status barely influenced the incremental QALYs. Figure 4c,d show the one-way sensitivity analysis for the transition probabilities. The tornado plot suggested that the probability of AIDS development (HIV status to AIDS status in both the with- and without-PrEP groups) and the infection probability (susceptible status to HIV status in the without-PrEP group) had the greatest influence on the incremental cost, whereas the influence of probability of death (natural, HIV, AIDS) was limited because they were small. Contrary, the transition probability of natural death had the most influence on the incremental QALYs.

**Figure 5:**
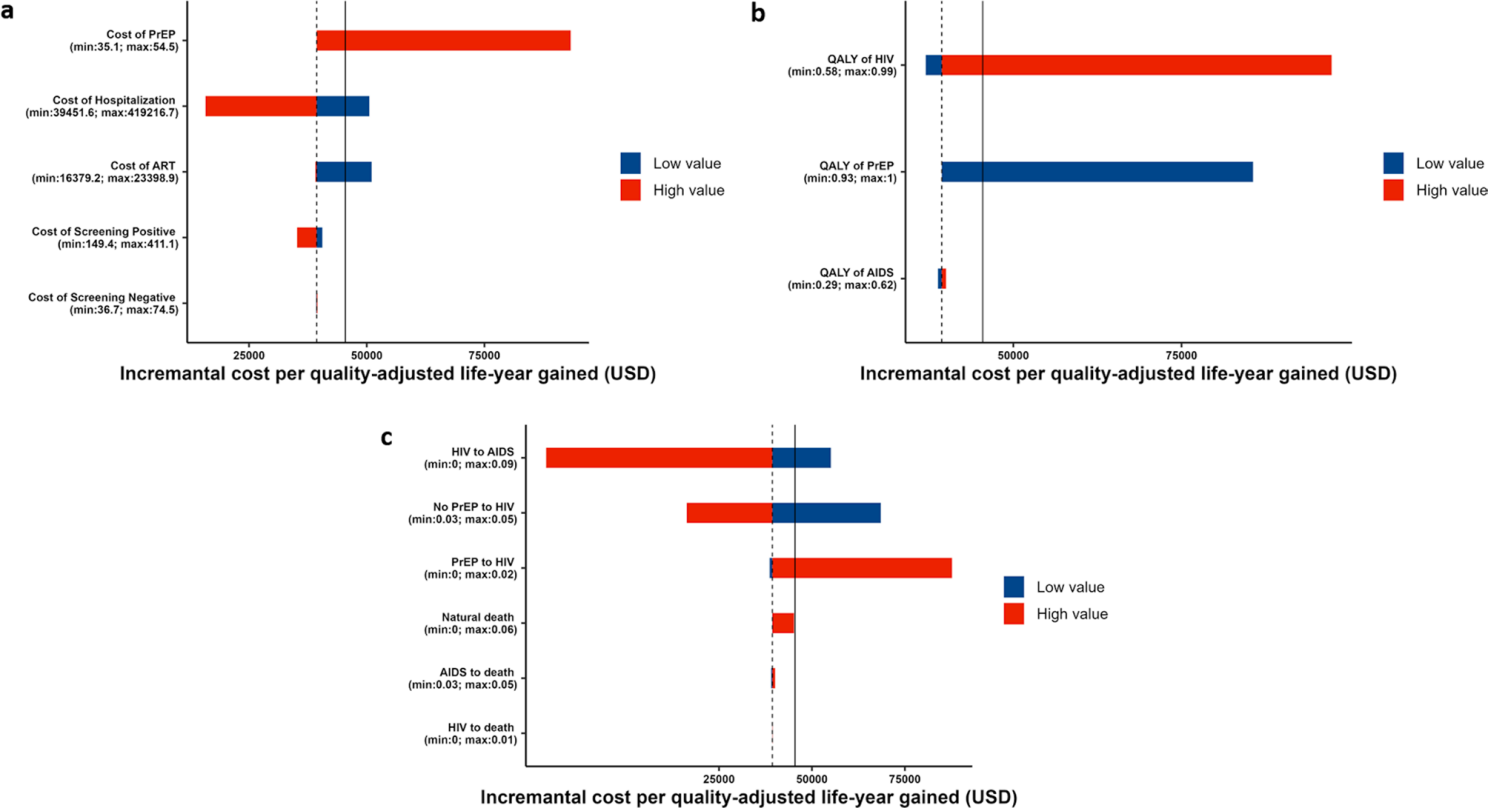
Cost-effectiveness evaluation of the pre-exposure prophylaxis (PrEP) program. The incremental cost per QALY gained (also known as incremental cost-effectiveness ratio [ICER]) in the 15 years since the introduction of the PrEP program with varied parameter values was calculated. Incremental cost varying each cost, QALYs, or transition parameter. The black solid vertical lines represent the cost-effectiveness threshold frequently referred to in Japan (5.0 million JPY, or 45,454.5 USD). The black dashed vertical lines represent the base case scenario (4.3 million JPY per QALY gained). Therefore, the values on the left of the base case scenario correspond to more favorable scenarios compared with baseline. Blue bars represent parameter values lower than baseline, and red bars represent parameter values higher than baseline. For example, when the cost of PrEP increased (red bar), the incremental cost also increased, which is not favorable. PrEP coverage was assumed to be 50%. (**a**) Cost parameters were varied: the cost of PrEP pills, cost of hospitalization due to AIDS, cost of ART for those infected by HIV, cost of screening for the HIV-positive population, and cost of screening for the HIV-negative population. (**b**) QALY parameters varied: QALYs for people with PrEP, people living with HIV, and AIDS. (**c**) The transition probabilities of the Markov model also varied. For the transition probability from PrEP to HIV, the PrEP efficacy was varied.

Figure 5 shows the tornado diagram of the parameters on the ICER for the 15-year time horizon. Figure 5a shows the one-way sensitivity analysis of the cost parameters. The cost of PrEP pills had the most influence on the ICER, whereas the cost of screening for susceptible and uninfected people and the cost of screening and health checkups for those with HIV barely influenced the ICER. As is intuitively imagined, costly PrEP pills increased the total cost and made the program less cost-effective. When the cost of the PrEP pills was increased to 54.5 USD per pill (highest value in the sensitivity range), the ICER increased to 93,636.4 USD from 39,090.9 USD at baseline. Given a cost-effectiveness threshold value of 45,454.5 USD (5.0 million JPY), the estimated cost-effectiveness threshold value for PrEP pills was 37.3 USD per pill. Figure 5b shows the one-way sensitivity analysis for the QALYs. The QALYs for HIV status had the most influence on the ICER, whereas the QALYs for AIDS status barely influenced the ICER. If lower QALYs were associated with PrEP use, the ICER increased substantially. Figure 5c shows the one-way sensitivity analysis for the transition probabilities. The tornado plot suggested that the probability of AIDS development (HIV status to AIDS status in both the with- and without-PrEP groups) and the infection probability (susceptible status to HIV status in the without-PrEP group) had the greatest influence on the ICER, whereas the influence of probability of death (natural, HIV, AIDS) was limited because they were small. Both a high infection probability in the group without PrEP that is caused by low PrEP efficacy and a high probability of AIDS development helped reduce total costs, making the program more cost-effective. In contrast, a high infection probability in the PrEP group increased the total cost and made the program less cost-effective because of the expense of PrEP pills and HIV treatment. In particular, the ICER increased to 68,181.8 USD for the lowest infection probability in the group without PrEP and to 87,272.7 USD for the highest infection probability in the group with PrEP in the sensitivity ranges.

**Figure 6:**
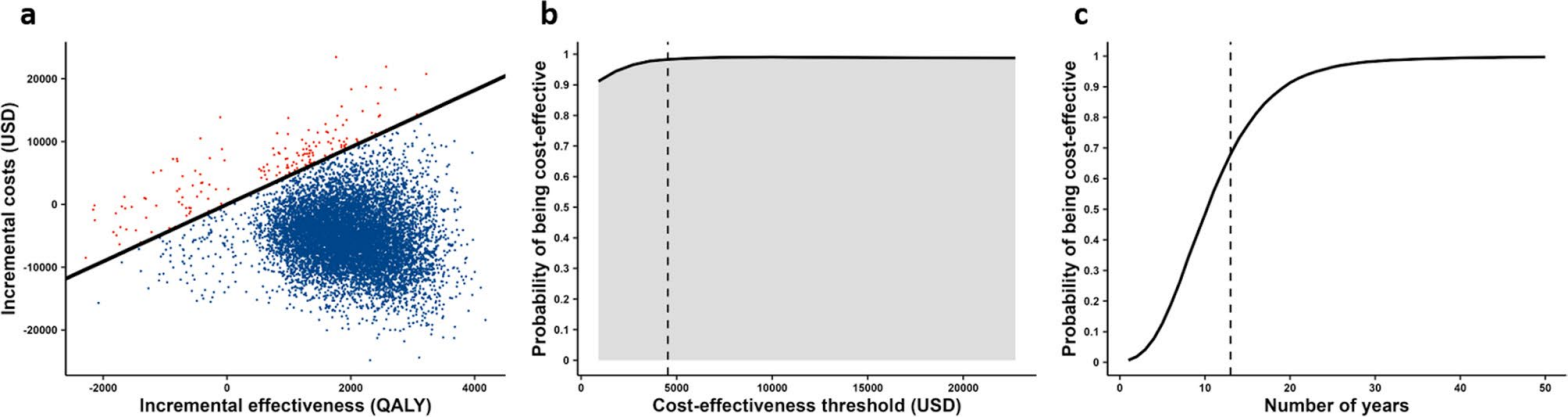
Probabilistic sensitivity analysis for the pre-exposure prophylaxis (PrEP) program. (**a**) Results of probabilistic sensitivity analysis. Each dot represents one simulation run. The *y*-axis represents the incremental costs of the PrEP program compared with no PrEP program. The *x*-axis represents incremental QALY with the PrEP program compared with no PrEP program. The black diagonal line represents cost per QALY gained of 5.0 million JPY. Therefore, the blue dots (below the line) are simulations with cost-effectiveness and the red dots (above the line) are simulations without cost-effectiveness. (**b**) The cost-effectiveness acceptability curve shows the percentage of simulations for which the PrEP program was more cost-effective compared with a scenario without the PrEP program at a cost-effectiveness threshold. (**c**) The change in probability of being cost-effective for the same set of parameters with the varied time horizon.

The cost-effectiveness plane (Fig. 6a) shows the uncertainty around our findings. We fixed the screening cost for both the HIV-positive and HIV-negative populations because we found they did not influence the ICER in the one-way sensitivity analysis. We also fixed the transition probabilities. The solid black line represents the willingness to pay (WTP), which was equivalent to the cost-effectiveness threshold value (45,454.5 USD/QALY). With this WTP value, the cost-effectiveness probability (probability that the ICER was above the cost-effectiveness threshold) was 98.3%. The cost-effectiveness acceptability curve (Fig. 6b) shows that cost-effectiveness probability increased with the cost-effectiveness threshold. Figure 6c shows the change in probability of being cost-effective for the same set of parameters with the varied time horizon. The probability of being cost-effective increases as the number of years since the program initiation increases.

**Figure 7:**
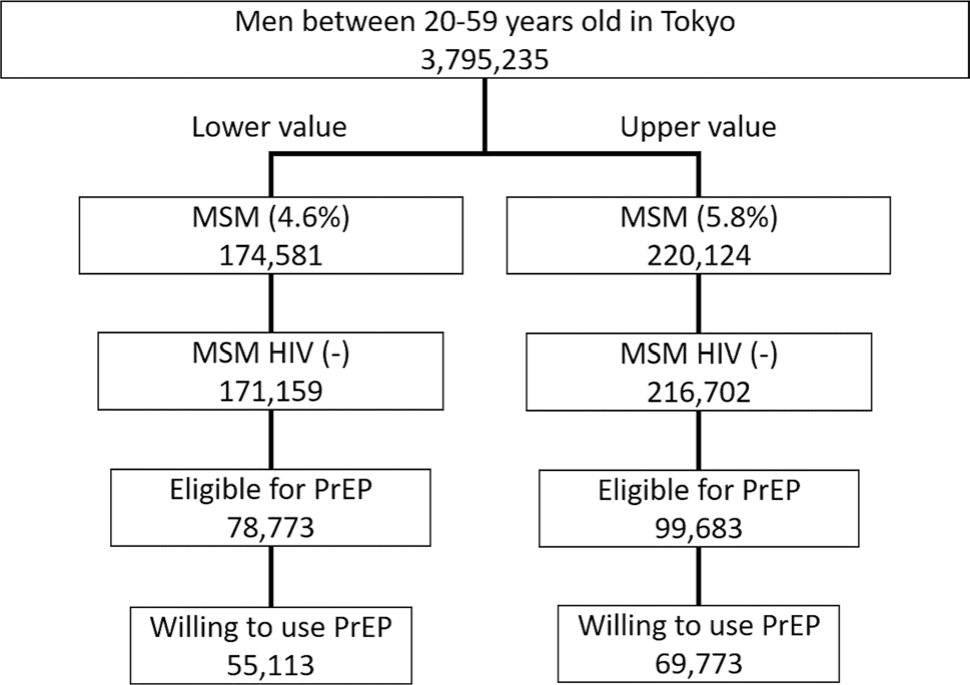
Estimation of the number of men who have sex with men (MSM) who are eligible for pre-exposure prophylaxis (PrEP) in Tokyo. The population denominator was based on the number of males aged 20–59 years in Tokyo. The left side represents the lower values of the number of MSM who meet each criterion and the right side represents the upper values.

For the budget impact analysis, we estimated that the total number of MSM in Tokyo who would be eligible for and willing to use PrEP ranged from 55,113 to 69,778 (Fig. 7). At current PrEP pill costs, the total annual cost (including screening costs) was 708.2–897.3 million USD per year if the intervention was on a daily basis.

## Discussion

Our results showed that the PrEP program would be cost-saving, even in an MSM cohort in Japan with a relatively low risk for HIV infection. Approving PrEP would result in health benefits and ultimately reduce the total costs relevant to HIV/AIDS prevention and infection at the population level. Once PrEP is approved and becomes prevalent in Japan, the cost will be reduced, and the PrEP program will become cost-effective more quickly.

We used a Markov model to describe HIV infection and disease progression to evaluate the cost-effectiveness of a PrEP program for MSM in Japan. The cost, utilities (measured by QALYs), and cost-effectiveness of the PrEP program were calculated from the simulated number of cases of HIV and AIDS over time. With 50% PrEP coverage, the cost-effectiveness measured by the ICER was 39,090.9 USD per QALY gained after 15 years, which was lower than the conventional cost-effectiveness threshold (45,454.5 USD/QALY). A one-way sensitivity analysis revealed that higher cost of PrEP pills and higher QALY for HIV may make the program less cost-effective. Developing cheaper PrEP pills may be important for a successful PrEP program.

Although PrEP is highly efficacious among MSM and is widely accepted in many countries, constructive discussion about the approval of PrEP in Japan has only occurred recently. We have conducted surveillance to understand how PrEP is viewed and would be used in the MSM population (N = 5120) in Japan. PrEP awareness among MSM in Japan has increased in the past few years, and 36.3% of MSM were aware of PrEP in 2018^23^. Although PrEP is not yet approved, some MSM (73 of 113) indicated that they had started using PrEP by privately importing ART drugs. Combined with our findings that PrEP is well accepted and that some in the MSM population have started using it, we believe our findings on the cost-effectiveness of a PrEP program support the approval of PrEP in Japan. Once approved, “on-demand” use of PrEP (not included in this study) could also be introduced in the population. Given the higher cost of daily PrEP, the cost-effectiveness may increase by introducing on-demand PrEP. PrEP approval will further reduce PrEP misuse without appropriate knowledge and instruction from health practitioners.

Several strengths of this study should be noted. First, to our knowledge, this is the first study to assess the cost-effectiveness of a PrEP program in an MSM cohort in Japan. Second, we performed one-way sensitivity and probabilistic sensitivity analyses to enable us to respond to uncertainty, including changes that may happen in the future. Third, we conducted budget impact analysis in addition to cost-effectiveness analysis. Considering the number of MSM, our budget impact analysis demonstrated a similar result to previous studies^24^. We found introduction of PrEP program in Japan was expected to cost 708.2–897.3 million USD; this means that PrEP is an affordable intervention in Japan because national medical expenses amount to over 363.6 billion USD^25^ and the cost of PrEP is likely to be covered by out-of-pocket expenses if it is approved.

This study also had several limitations that should be noted. First, we used a static decision-analytic model instead of a dynamic transmission model. Dynamic models are widely used to describe epidemiologic dynamics in populations where the infection probability changes over time, depending on the number of infected people. In contrast, the infection probability was assumed to be static in the Markov model used in this study. In Japan, HIV incidence has been stable in the past 10 years, so we believed that this assumption was acceptable. Further, because we assumed a small MSM cohort, the impact of the PrEP program on the MSM population (and therefore on incidence) was limited^26^. If the indirect benefits of PrEP (i.e., not contracting HIV means no possibility of transmitting HIV) were incorporated, ICER would be lower because PrEP also increased QALYs for people who were not using PrEP. Second, data for QALYs were not available for this targeted population, and we used yearly QALYs estimated in other high-income countries^27–30^. Our results should be interpreted with caution because we had no empirical QALY data for patients with HIV in Japan, and the QALY value of HIV may affect the results in the short term. Third, we did not assume that PrEP caused change in risk behavior, known as risk compensation. We also assumed the adherence to PrEP was 100%. Our pilot cohort study (details of the cohort study are available in https://rctportal.niph.go.jp/en/detail?trial_id=UMIN000018699 [Registry number: UMIN000018699].) showed that people who took PrEP were well aware of the risk compensation and adhered to PrEP as prescribed. If PrEP users have more risky sex, cost-effectiveness would be negatively affected because of the increasing cost related to managing non-HIV sexually transmitted infections (STIs). However, risk compensation was not prevalent at a population level. For example, a previous modeling study found that PrEP coverage resulted in a decline in the incidence of STIs^31^. Fourth, we assumed that the population was a homogeneous group of MSM. The PrEP coverage was also assigned homogeneously to the PrEP group throughout the time horizon. Although this was not the most realistic way to model, we believed that it was reasonable to assume immediate scale-up of PrEP coverage (0%, 50%, or 100%) in the homogeneous group, which was split into with-PrEP and without-PrEP groups as we were investigating a cohort.

In conclusion, the introduction of a PrEP program for an MSM cohort in Japan is cost-saving, at least in the long term. Cost-effectiveness assessment should be updated to reflect the cost of PrEP pills, which substantially impact the cost-effectiveness. A challenge for further research is to estimate actual QALYs of Japanese patients with HIV with empirical data to make the results more reliable.

## Methods

### HIV/AIDS infection and disease progression model (Markov model)

A discrete-time Markov chain with stationary transition probabilities was used to describe HIV infection and disease progression in the MSM cohort. The cohort size was assumed to be 1,000. The model comprised four different health status groups of infection and disease progression: susceptible and uninfected (denoted by $S$), HIV-infected without AIDS ($HIV$), HIV-infected with AIDS ($AIDS$), and dead ($D$). All individuals were categorized into one of these groups. The four health conditions were then further annotated by PrEP. Therefore, two independent Markov models were prepared for populations with and without PrEP (Fig. 1a). Delay in the diagnosis of HIV and AIDS was not considered in this cohort; therefore, the health status groups corresponded to diagnosed status, and the treatment was initiated immediately after diagnosis. Status update was performed every year. Note that although PrEP decreased the infection probability, it did not influence disease progression in this simulation. The simulation was run with different PrEP coverages (i.e., 0%–100%). We performed 1,000 simulations for each PrEP coverage.

### Cost-effectiveness analysis

The financial cost (i.e., opportunity cost is not considered) was evaluated from the perspective of the healthcare payer, based on the number of individuals in each status in each year obtained from the simulation. Specifically, different costs and QALYs were assigned to each health status in the Markov model (Table 1). Susceptible and uninfected individuals (${S}_{p}$ and ${S}_{n}$) in the cohort need to be screened for HIV and STIs every 3 months (therefore, the yearly cost was derived by multiplying the screening cost by four). Those susceptible in the PrEP group (${S}_{p}$) received daily PrEP pills (tenofovir disoproxil fumarate and emtricitabine). Those with HIV ($HI{V}_{n}$, $HI{V}_{p}$, $AID{S}_{n}$, and $AID{S}_{p}$) needed to be screened for non-HIV STIs and receive health checkups and ART. Those who developed AIDS ($AID{S}_{n}$ and $AID{S}_{p}$) might be hospitalized because of complications. Monthly hospitalization costs were calculated as a product of the risk for hospitalization due to AIDS per month, and the mean cost of hospitalization per month was estimated using data obtained in Japan. The utilities used to calculate the QALYs considered reduced or increased quality of life. Therefore, effectiveness was evaluated based on the total QALYs in each year. The total QALYs were calculated by summing the yearly QALYs for all 1,000 individuals in the cohort. Both costs and QALYs were discounted annually by 2.0%^42^.

**Table 1:** Parameter values for the Markov model and cost-effectiveness analysis.

Parameter^†^	Baseline value	Sensitivity range***
**Transition probability per year and PrEP efficacy**		
Infection probability for those without PrEP (*S*_*n*_ to *HIV*_*n*_)	0.038^20^	0.026–0.054^32^
Probability of dying from Non-HIV/AIDS-related causes (*S*_*n*_ to *D*_*n*_ and *S*_*p*_ to *D*_*p*_)	0.0014^23,33^	0.0003–0.0610^23,33^
PrEP efficacy for reducing infection probability^‡^	0.99^34,35^	0.63–1.00^32^
Probability of AIDS development (*HIV*_*n*_ to *AIDS*_*n*_ and *HIV*_*p*_ to *AIDS*_*p*_)	0.013^36^	0.000–0.087^36^
Probability of dying due to HIV infection (*HIV*_*n*_ to *D*_*n*_ and *HIV*_*p*_ to *D*_*p*_)	0.0051^37^	0.0042–0.0066^37^
Probability of dying due to AIDS (*AIDS*_*n*_ to *D*_*n*_ and *AIDS*_*p*_ to *D*_*p*_)	0.034^38^	0.027–0.049^38^
**Costs (in USD)**		
PrEP pills for *S*_*p*_^§^	35.1/pill^39^	35.1–54.5^39^
Screening for HIV and other STIs in the HIV-negative population (*S*_*p*_ and *S*_*n*_) ^¶^	52.4/visit^39^	36.7–74.5^39^
ART treatment for *HIV*_*n*_, *HIV*_*p*_, *AIDS*_*n*_, and *AIDS*_*p*_	1,936.7/person-month^40^	1,364.9–1,949.9^40^
Screening for STIs except HIV and health checkups in the HIV-positive population (*HIV*_*n*_, *HIV*_*p*_, *AIDS*_*n*_, and *AIDS*_*p*_)*	207.6/visit^39,40^	149.4–411.1^39,40^
AIDS hospitalization for *AIDS*_*n*_ and *AIDS*_p_**	13,454.5/person-month^39,41^	3,287.6–34,934.7^39,41^
**Annual QALYs**		
*S*_*p*_	1.0	0.93–1.0^27^
*S*_*n*_	0.95	0.94–0.96^27^
*HIV*_*n*_ and *HIV*_*p*_	0.62^28^	0.58^29^–0.99^30^
*AIDS*_*n*_ and *AIDS*_*p*_	0.45^14^	0.29–0.62^14^
*D*_*n*_ and *D*_*p*_	0.0	

PrEP, pre-exposure prophylaxis.

^†^Transition probabilities are per year and costs are per month.

^‡^The transition probability (*S*_*p*_ to *HIV*_*p*_) is the product of the transition probability (*S*_*n*_ to *HIV*_*n*_) and 1-(PrEP efficacy).

^§^ Cost of PrEP pills per month calculated as a product of PrEP pill cost (per pill) and the number of pills per month (30 pills).

^¶^Screening for susceptible and uninfected individuals in the cohort (*S*_*p*_, *S*_*n*_) includes the test for HIV and other sexually transmitted infections (STIs) performed every 3 months. Monthly screening cost was derived by dividing the screening cost every 3 months by 3.

*HIV-positive people (*HIV*_*n*_, *HIV*_*p*_, *AIDS*_*n*_, and *AIDS*_*p*_) need to take a screening test for STIs except HIV and have a health checkup.

**The cost of AIDS hospitalization (per month) calculated as a product of the probability of hospitalization for *AIDS*_*n*_ and *AIDS*_*p*_ per month and the mean cost of hospitalization per month.

***Sensitivity ranges were used for the one-way sensitivity analysis.

The incremental cost and the incremental QALYs over a 30-year period were used to assess the cost-effectiveness on the program. The ICER was also used to assess the cost-effectiveness of the program over a 15-year period. The ICER is a quotient of incremental cost (Δcost: total cost with the program minus total cost without the program) and incremental utilities (ΔQALY: total QALYs with the program minus that without the program). The model parameters, including the transition probabilities, costs (PrEP pills, treatment, screening, and hospitalization), and PrEP efficacy are summarized in Table 1. The transition probabilities were estimated from observed counts for movements between health status groups in Japan. The transition probability ($${S}_{p}$$ to $$HI{V}_{p}$$) is the product of the transition probability ($${S}_{n}$$ to $$HI{V}_{n}$$) and $$1-($$ PrEP efficacy $$)$$. The values for cost and PrEP efficacy were obtained from literature relevant to Japan. We determined whether introducing the PrEP program was cost-effective by comparing the calculated ICER with the cost-effectiveness threshold frequently referred to in Japan (5.0 million JPY or 45,454.5 USD)^43^.

### Budget impact analysis

To estimate the number of MSM in Tokyo who may use PrEP, we based the population denominator on the Statistic Bureau of Japan 2015^44^. Ichikawa et al.^45^ estimated the proportion of MSM in Tokyo as 5.8%, and the proportion of MSM in Japan as 4.6% (95% confidence interval:4.4–4.8%). We therefore used 5.8% as the upper value and 4.6% as the lower value for the proportion of MSM in Tokyo. We then subtracted 3,422 of MSM who were living with HIV. In the questionnaire about PrEP^23^ for MSM in Japan, 46.0% answered that they received routine examination for HIV and other STIs. Because medical checkups are required when taking PrEP, we used this proportion as the proportion eligible for PrEP. We also calculated the proportion eligible for PrEP using criteria used in other countries^24,46,47^. Using data from the same survey, we assumed that 70.0% of these men were willing to use PrEP. We then estimated the total cost for providing PrEP to the estimated eligible population. We included the costs of PrEP pills in Japan in the calculation as well as the screening costs associated with PrEP follow-up. Other indirect costs were not included.

### Sensitivity analyses

We performed a one-way sensitivity analysis for the cost-effectiveness on cost, QALY parameters and transition parameters by setting the highest or lowest value of a single parameter in the sensitivity range while fixing others at baseline. The PrEP coverage was fixed (50%). The results from the one-way sensitivity analysis were summarized in a tornado diagram to identify the parameters with large impacts on the cost-utility results in the realistic range.

Furthermore, a probabilistic sensitivity analysis using Monte-Carlo simulation was performed to assess the probability that the program would become cost-effective. The cost, QALY parameters, and PrEP coverage were randomly sampled from the estimated distributions. The parameter distributions were assumed to be independent and identically distributed. Gamma distributions were assumed for the distributions of cost parameters, lognormal distributions or PERT (program evaluation review technique) distribution were assumed for annual QALYs with constraints between 0 and 1, and a beta distribution was assumed for PrEP coverage^15,22,39–41,48^. A total of 10,000 simulations were performed for the probabilistic sensitivity analysis. All data were obtained from literature published between September 2019 and February 2021. All statistical data were analyzed using the statistical software R, version 3.5.3 (R Core Team, Vienna, Austria, 2017). All methods were carried out in accordance with relevant guidelines and regulations.

### Ethical statement

This research involved no identifiable data, and ethics approval was not required.
